# Recent Progress of Perovskite Nanocrystals in Chem/Bio Sensing

**DOI:** 10.3390/bios12090754

**Published:** 2022-09-14

**Authors:** Dailu Jia, Meng Xu, Shuang Mu, Wei Ren, Chenghui Liu

**Affiliations:** 1 Key Laboratory of Applied Surface and Colloid Chemistry, Ministry of Education, Xi’an 710119, China; 2 Key Laboratory of Analytical Chemistry for Life Science of Shaanxi Province, Xi’an 710119, China; 3 School of Chemistry & Chemical Engineering, Shaanxi Normal University, Xi’an 710119, China

**Keywords:** perovskite nanocrystals, instability, water incompatibility, chem/bio sensing

## Abstract

Perovskite nanocrystals (PNCs) are endowed with extraordinary photophysical properties such as wide absorption spectra, high quantum yield, and narrow emission bands. However, the inherent shortcomings, especially the instability in polar solvents and water incompatibility, have hindered their application as probes in chem/bio sensing. In this review, we give a fundamental understanding of the challenges when using PNCs for chem/bio sensing and summarize recent progress in this area, including the application of PNCs in various sensors and the corresponding strategies to maintain their structural integrity. Finally, we provide perspectives to promote the future development of PNCs for chem/bio sensing applications.

## 1. Introduction

Since the discovery of the perovskite calcium titanate (CaTiO_3_) by Gustav Rose in 1839, perovskites have attracted wide attention from researchers. In the most recent 10 years, perovskite nanocrystals (PNCs) have been studied extensively due to their excellent optical and electrical properties [1]. The general chemical formula of PNCs is ABX_3_, in which A (such as Cs^+^, methylammonium [CH_3_NH_3_^+^, MA], formamidinium [(CH(NH)_2_)_2_^+^, FA]) and B (Pb^2+^, Sn^2+^, Ge^2+^, Bi^3+^, In^3+^ or Sb^3+^) are cations, and X (X = Cl, Br, I) represents anions that octahedrally coordinate to B. The large A-site cations and the smaller B-site cations allow [BX_6_]^4−^ octahedra to corner-share in a 3D framework, with the A-site cations located in the framework cavities [2]. Until now, researchers have developed effective synthetic methods to obtain PNCs, including high-temperature injection [3], room-temperature reprecipitation [4], microwave method [5], solvothermal synthesis [6], ultrasonication [7], and chemical vapor deposition method [8], etc.

At present, due to their size-dependent luminescence, narrow emission bands, high photoluminescence quantum yields (PLQYs), high defect tolerance, and excellent charge transport properties [3,9,10,11,12], PNCs have been widely used in solar cells [13,14], light-emitting diode (LED) [15,16], lasers [17,18], photodetectors [19,20], and other optical devices [21,22]. Despite the great potential shown, the chem/bio sensing applications of PNCs remain challenging due to their poor structural stability. It is because they are ionic crystals composed of ionic bonds. This character makes them easily react with air, moisture, light, and heat, leading to degradation if they are not stored well [23]. Thus, stability is the predominant challenge of PNCs in chem/bio sensing applications. Fortunately, in recent years, researchers have developed a series of strategies to overcome such drawbacks of PNCs and applied them in the sensing of a range of target molecules, including gases [24,25], metal ions [26,27,28], and biomolecules [29,30,31], etc. In this review, we provide readers with the current progress of PNCs in chem/bio sensing, mainly about their applications as sensing probes in various scenarios and the detailed techniques to improve their stability and bio-compatibility. At the end of this review, we point out that the development of material technologies, such as synthesizing high-stability PNCs, lead-free PNCs, hybrid PNCs nanomaterials, as well as the advancement of novel PNCs-based sensor fabrication technologies will be the future trends to lead the chem/bio sensing applications of PNCs.

## 2. PNCs in Chem/Bio Sensing Applications

**Figure 1:**
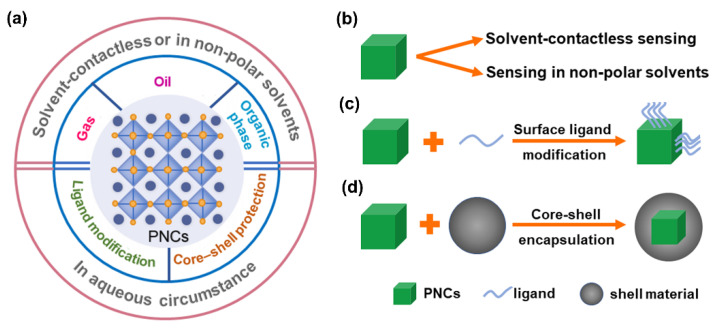
(**a**) Different chem/bio sensing application scenarios of PNCs. (**b**) Directly use PNCs as sensing probes for solvent-contactless sensing and sensing in non-polar solvents. (**c**) Ligand modification strategy and (**d**) core-shell encapsulation strategy for protecting PNCs towards the sensing applications in aqueous circumstances.

Considering the inherent instability of PNCs, their current chem/bio sensing applications can be classified into two categories. On the one hand, the as-synthesized PNCs can be directly applied in the sensing in solvent-contactless manners or in non-polar solvents, such as target analysis in the gas/oil/organic phase. In these situations, the crystal structure and chemical composition of PNCs remain stable in the sensing environments, while the target molecules will lead to the change of the PNCs’ optical properties via different routes, such as crystal structure degradation and halogen exchange. On the other hand, aiming at using PNCs as sensing probes in “harsh” aqueous circumstances, various protection methods have been designed and developed to physically isolate PNCs from the outside environment and provide a substrate for further surface functionalization. Generally, the protection methods can be classified as ligand modification and core-shell encapsulation (Figure 1). Accordingly, in the following parts of this review, we will introduce the progress achieved in the chem/bio sensing applications of PNCs.

### 2.1. PNCs Directly Employed as Fluorescent Probes

PNCs are endowed with high PLQYs and strong fluorescence intensity with tunable wavelength, which hold the potential to be used as probes for rapid and sensitive fluorescent chem/bio sensing. However, due to the inherent ionic feature, PNCs are extremely susceptible to moisture and some gases. This remains a major obstacle to their chem/bio sensing applications. From another perspective, this in turn gives researchers a chance to apply PNCs in some specific sensing areas, such as solvent-contactless sensing (i.e., humidity sensing and gas sensing) and sensing in non-polar solvents.

#### 2.1.1. Humidity Sensing

**Figure 2:**
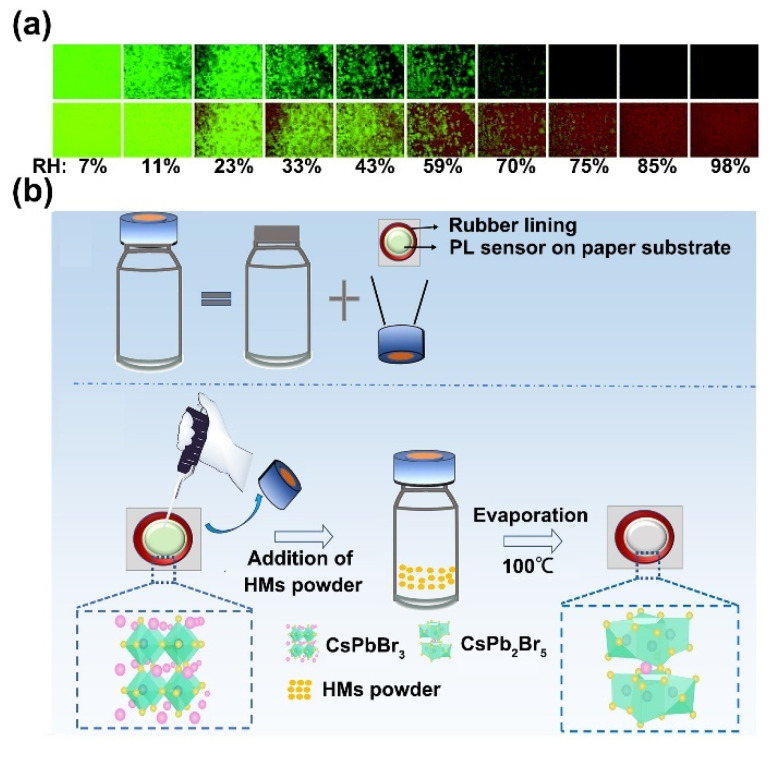
(**a**) Color responses of the PNC humidity sensor toward different RH ranging from 7 to 98% without TFPP (top panel) and with TFPP (bottom panel) at room temperature. Reprinted from [32], with permission from Royal Society of Chemistry. (**b**) The device for detecting water content in herbal medicines (denoted as HMs in the figure) based on CsPbBr_3_ PNCs. Reprinted from [33], with permission from Elsevier.

It has been well known that the crystal structure of PNCs can be easily destroyed by a trace amount of water due to their ionic nature. The high instability of PNCs in the humid environment gives researchers a hint that PNCs might be effective probes for humidity analysis. For example, Chen et al. reported a sensitive and reversible humidity sensor by using CH_3_NH_3_PbBr_3_ PNCs. This kind of PNCs showed bright fluorescence at 530 nm, and the fluorescence was effectively quenched when the PNCs were exposed to moisture (top panel of Figure 2a). Furthermore, by coupling the PNCs with a red-fluorescence reference dye, 5,10,15,20-tetrakis(pentafluorophenyl)porphyrin (TFPP), a colorimetric relative humidity (RH) sensor was developed. Specifically, PNCs and TFPP were embedded into a layer of polystyrene to build an RH-sensing film. By using TFPP as a stable internal reference to provide invariable red fluorescence, the sensor displayed an obvious color change from green to brown with the increased RH content to quench the green fluorescence of the PNCs (bottom panel of Figure 2a) [32]. In another work by Fu et al., a novel fluorescence paper sensor that used CsPbBr_3_ PNCs for the rapid detection of water content in herbal medicines with a turn-off mode was reported. To guarantee the sensing accuracy, the authors designed a very facile evaporative device, which had a CsPbBr_3_-covered paper substrate contained in a glass vial as the fluorescence signal transducer (Figure 2b) [33].

Taking the benefit of the high sensitivity to moisture, PNCs can be adopted to build up humidity sensors. However, the PNC-based humidity sensors still suffer from some drawbacks, such as the use of toxic metal ions and storage challenges.

#### 2.1.2. Gas Sensing

**Figure 3:**
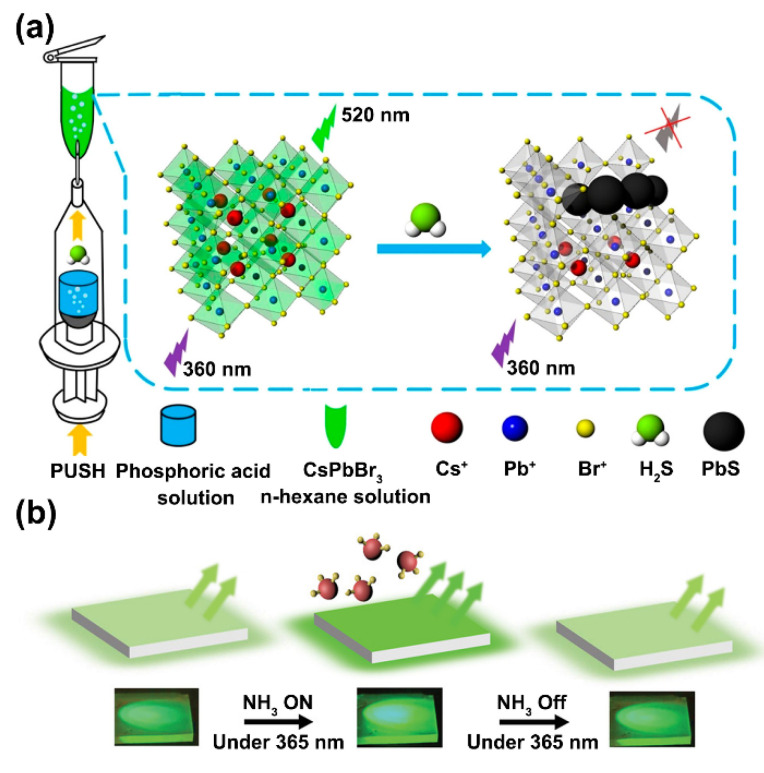
(**a**) Schematic mechanism of H_2_S gas detection by using CsPbBr_3_ PNCs. Reprinted from [34], with permission from American Chemical Society. (**b**) Schematic diagram of CsPbBr_3_ PNC film sensor for the detection of NH_3_ gas. Reprinted from [36], with permission from John Wiley and Sons.

Besides humidity/water sensing, gas sensing is another area suitable for PNC probes because it can facilely avoid the obstacle of the poor stability of PNCs in liquid samples. Recently, Lin et al. reported a novel fluorescent sensor for the rapid detection of H_2_S gas. In this work, a simple device was established to separately store the CsPbBr_3_ PNCs n-hexane solution in a centrifuge tube and the phosphoric acid solution in an injector, in which the centrifuge tube adhered to the needle of the injector. This protected the PNCs from contacting water. Due to the poor solubility of H_2_S in water, when injecting the hydrogen sulfide sample into the phosphoric acid solution by using a microliter syringe, almost all the hydrogen sulfide escaped from the aqueous solution and then passed into the n-hexane solution to react with the PNCs. In this process, H_2_S passed through the oleic acid (OA) and oleylamine (OAm) ligands on the surface of the PNCs to reach the inside of PNCs and reacted with Pb^2+^, forming more stable PbS nanoparticles, which led to structural destruction of the PNCs and fluorescence quenching. Therefore, the system’s fluorescent intensity was negatively correlated to the H_2_S content. This sensor displayed a linear relationship in the range of 0–100 μM with a limit of detection (LOD) of 0.18 μM and was adequate to measure the H_2_S content in rat brain samples (Figure 3a) [34]. Ma et al. developed a rapid gaseous anion-exchange method to detect HCl vapor by using CsPbBr_3_ PNCs as probes. In this study, the CsPbBr_3_ PNCs were drop-casted on glass substrates or filter paper. The HCl vapor was produced by the reaction between H_2_SO_4_ (98%) and NaCl. The anion-exchange reactions were conducted in an air-tight container, in which the PNCs on the supports were exposed to various amounts of HCl vapor for detection. After Cl/Br halogen-exchange, the resultant CsPb(Br/Cl)_3_ PNCs showed a significant blue-shift in the fluorescence spectra [35]. Dong et al. designed a CsPbBr_3_ PNCs film sensor for the detection of NH_3_ gas. They found that NH_3_ gas dramatically increased the fluorescence intensity of CsPbBr_3_ because ammonia passivated the surface defects of PNCs even if there was no chemical reaction, which enabled the fluorescence to change reversibly without damaging the PNC structure. The turn-on sensor achieved a LOD and a linear range of 8.85 ppm and 25–350 ppm, respectively (Figure 3b) [36].

According to these works, PNCs are promising candidates for the sensing of various gases. Nevertheless, owing to the relatively poor stability of PNCs, the interference of other gaseous content, such as water vapor in the gas samples, can hardly be eliminated in practical applications. Therefore, it is supposed that great efforts should be devoted to integrating PNCs with portable gas separation devices, and developing highly specific target-responsive protection techniques to improve the stability and specificity of the PNC-based gas sensors.

#### 2.1.3. Sensing in Non-Polar Solvents

**Figure 4:**
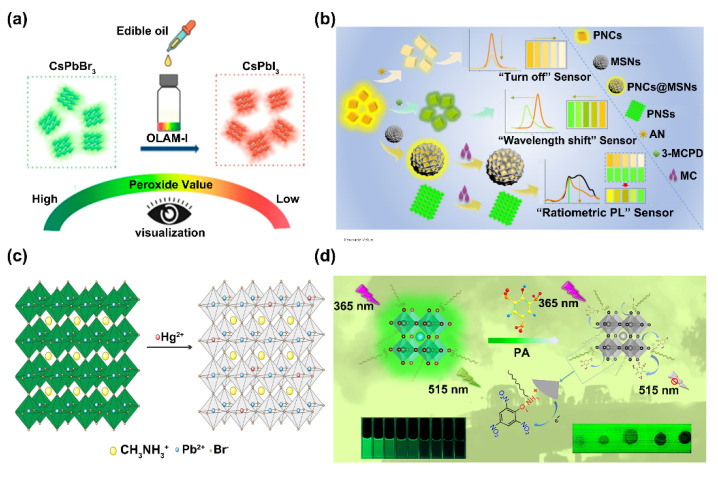
(**a**) Sensing mechanism of the wavelength-shift-based colorimetric method for the peroxide number determination of edible oil by using CsPbBr_3_ PNCs. Reprinted from [38], with permission from American Chemical Society. (**b**) Schematic illustration of multi-mode sensing of AN and 3-MCPD in edible oil. Reprinted from [40], with permission from American Chemical Society. (**c**) The illustration of the ion exchange process between the CH_3_NH_3_PbBr_3_ PNCs and Hg^2+^ for Hg^2+^ sensing. Reprinted from [41], with permission from Elsevier. (**d**) Schematic of the sensitive fluorescence detection of PA by using CsPbBr_3_ PNCs. Reprinted from [44], with permission from Elsevier.

Solvent-contactless humidity sensing and gas sensing can directly avoid the damage of PNCs by detrimental solvents. Meanwhile, target sensing in non-polar solvents is another application scenario to minimize the non-specific structural damage of PNCs. For instance, oil is a typical kind of non-polar solvent. In 2017, Xu et al. reported that the colloidal CsPbX_3_ PNCs possessed a remarkable probing ability for metal ions, especially for high sensitivity and selectivity Cu^2+^ ion detection. They reported that as the concentration of Cu^2+^ increased, the fluorescence intensity of the CsPbBr_3_ PNCs monotonically decreased. The attractive phenomenon was attributed to the adsorption of the Cu^2+^ ions to the surface of CsPbBr_3_ PNCs. This process was so fast that the equilibrium of a stable fluorescence was reached within seconds. Thus, the CsPbBr_3_ PNCs were successfully applied to rapidly probing Cu^2+^ ions in vehicle-lubricating oils and edible oils [37]. After then, Chen et al. developed a wavelength-shift-based colorimetric method for peroxide number determination of edible oil by using CsPbBr_3_ PNCs. They found that the fluorescence emission wavelength of CsPbBr_3_ PNCs was gradually red-shifted via halide exchange with the dropwise addition of oleylammonium iodide (OLAM-I). Correspondingly, the color changed from green to yellow and finally to red with the addition of OLAM-I. Therefore, the different emission wavelengths/colors in the detection system represented the peroxide numbers in an edible oil sample. Taking advantage of the halogen exchange feature of the PNCs and the redox reaction between OLAM-I and the peroxides in edible oil, a colorimetric sensor was built for the determination of the peroxide number of edible oil samples (Figure 4a) [38]. Afterward, Feng et al. proposed a novel fluorescent sensor for the rapid analysis of total polar materials (TPM) in edible oils by employing CsPbBr_3_ PNCs. As the content of TPM increased, the fluorescence intensity of the PNCs was quenched sequentially. The quenching effect was revealed in olive oil, soybean oil, and sunflower oil. Moreover, a paper-based CsPbBr_3_ PNC fluorescent sensor was established for the real-time determination of TPM content [39]. Furthermore, Zhao et al. developed a multi-mode PNC-based sensor for monitoring acid number (AN) (“turn-off” fluorescence sensor) and 3-chloro-1,2-propanediol (3-MCPD) (“wavelength-shift” colorimetric sensor) in edible oil. In this work, oil-soluble CsPbBr_1_._5_I_1_._5_ PNCs were prepared and used to detect AN with a “turn-off” fluorescence sensing mode depending on the acid-sensitive fluorescence quenching. Meanwhile, the “wavelength-shift” colorimetric sensing for 3-MCPD detection relied on the halogen exchange between CsPbBr_1_._5_I_1_._5_ PNCs and Cl elements of 3-MCPD (Figure 4b) [40].

Besides oil, PNCs can also be applied to analyte detection in other non-polar organic solvents. As an example, based on the ion exchange strategy, Tian et al. designed a fluorescence sensor for the visual detection of Hg^2+^ in toluene by using CH_3_NH_3_PbBr_3_ PNCs as probes. The strong green fluorescence of CH_3_NH_3_PbBr_3_ PNCs was dramatically quenched after Pb^2+^/Hg^2+^ ion exchange (Figure 4c) [41]. Aside from ion exchange, electron transfer has become one of the most frequently used mechanisms in PNCs-based fluorescent sensing. The tunable emission peak and high PLQY of PNCs make them suitable to act as electron donors in an electron transfer system. For example, based on the effective electron transfer from the PNCs to Cu^2+^, Zhu et al. reported a turn-off sensor for the selective detection of Cu^2+^ in hexane by using CsPbBr_3_ PNCs as the probes. In this system, the fluorescence of CsPbBr_3_ PNCs was significantly quenched within several seconds after the addition of Cu^2+^ [42]. On account of electron transfer, Nair et al. used CH_3_NH_3_PbBr_3_ PNCs to detect 2,4,6-trinitrophenol (TNP, picric acid) in toluene. In their design, the hydroxyl group of TNP formed stable hydrogen bonds with PNCs. This interaction brought the TNP close to the PNCs, resulting in fluorescence quenching. Therefore, the fluorescent intensity of the PNCs was negatively correlated to the concentration of TNP [43]. In another work by Wang et al., taking advantage of the formation of electrostatic complex and the electron transfer between picric acid (PA) and PNCs, a facile fluorescence turn-off approach was established by using high fluorescence efficiency CsPbBr_3_ PNCs to detect trace concentrations of PA in organic solution, the LOD of which could be as low as 0.8 nM (Figure 4d) [44].

Because PNCs can retain their structural integrity in non-polar solvents, they can be directly applied to the detection of target molecules in such environments without extra surface modification. Although such kinds of sensors are of high sensitivity, the target species are quite limited. For example, almost all the biological targets are hydrophilic and exist in the aqueous phase that is not compatible with pristine PNCs. Thus, to broaden the chem/bio sensing application scenario of PNCs, especially sensing in aqueous phases, it is of paramount significance to develop surface modification or encapsulation strategies to prevent the PNCs from being destroyed in polar solvents.

### 2.2. Surface Ligand Modification for Aqueous Phase Sensing

Currently, the as-synthesized PNCs can only be directly used in analyzing the target molecules in non-polar solvents or analyzing the gas/H_2_O targets in a solvent-contactless manner. To further expand the application scope of PNCs to the chem/bio sensing in polar solvents, e.g., in biocompatible water solutions, surface engineering on PNCs is inevitable. Among the PNC surface engineering methods, ligand modification seems to be the most convenient one toward the sensing applications.

#### 2.2.1. Small Amphiphilic Ligand Coating

**Figure 5:**
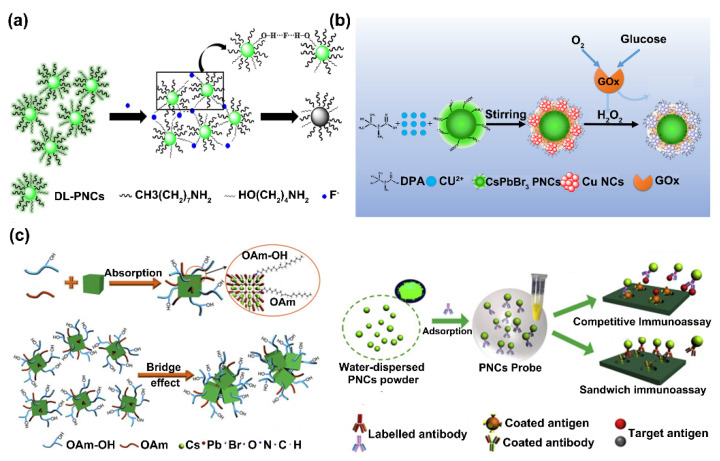
(**a**) Schematic illustration of the F^−^ sensing mechanism by using dual ligand (DL)-PNCs. Reprinted from [45], with permission from Elsevier. (**b**) Schematic depicting the CsPbBr_3_@Cu probe preparation and ratiometric detection of glucose. Reprinted from [46], with permission from American Chemical Society. (**c**) Schematic for the CsPbBr_3_ PNCs-based fluorescence-linked immunosorbent assay. Reprinted from [47], with permission from Elsevier.

Biocompatible small amphiphilic ligand modification methods have been developed to improve the water compatibility and fluorescence stability of PNCs for chem/bio sensing applications. For example, Lu et al. developed a fluoride (F^-^)-responded CH_3_NH_3_PbBr_3_ PNC probe by using 6-amino-1-hexanol (AH) and n-octylamine as dual ligands to avoid the aggregation and improve the stability of the PNCs. When F^−^ was present, the hydrogen bonding between the hydroxyl group of AH and F^−^ induced the growth and fluorescence quenching of the PNCs. Ultimately, the LOD was down to 3.2 μM, which was much lower than the WHO guideline (Figure 5a) [45]. Du et al. constructed a ratiometric fluorescent sensor for glucose assay through hybridizing green emission CsPbBr_3_ PNCs and red emission copper nanoclusters. In this sensing, D-penicillamine was used as the stabilizing agent of CsPbBr_3_. According to their design, glucose produced H_2_O_2_ under the catalysis of glucose oxidase (GOx), which quenched the fluorescence of copper nanoclusters at 645 nm, whereas it had no obvious influence on the fluorescence of CsPbBr_3_ PNCs at 517 nm, enabling the ratiometric detection of glucose (Figure 5b) [46]. Zeng et al. reported a sensitive perovskite fluorescence-linked immunosorbent method for aflatoxin M1 (AFM1) and carcinoembryonic antigen (CEA) detection by using CsPbBr_3_ PNCs. In this work, oleylamine (OAm) and oleylamine-OH (OAm-OH) were adopted as the surface ligand molecules to maintain the stability and water dispersibility of CsPbBr_3_. After this treatment, the PNCs functionalized with hydrophilic hydroxyl groups achieved a water dispersion of 3.4 mg/mL. Consequently, a quantifiable PNC-based fluorescence-linked immunosorbent methodology was constructed that possesses both competitive immunoassay and sandwich immunoassay capabilities. For the competitive immunoassay, the coating antigen (AFM1-BSA) was immobilized in each well of a 96-well microtiter plate, and the target antigen (AFM1) and the PNCs-AFM1 antibody probe were presented in the solution. Then, AFM1 and AFM1-BSA competed to combine with the PNCs-AFM1 probes. Finally, the fluorescence intensity decreased as the concentration of AFM1 increased. For the sandwich immunoassay, the PNCs-CEA antibody probe was captured by the target (CEA) and coated CEA antibody to form a sandwich structure. Thus, the intensity of fluorescence increases with a higher concentration of CEA after washing (Figure 5c) [47]. Lee et al. proposed a fluorescence sensor to detect tetracycline (TC) in food samples based on the inner filter effect between TC and Cs_4_PbBr_6_/CsPbBr_3_ PNCs. In their design, the PNCs were protected by perofluorooctyltriethyloxylsilane (PFOS) fluorocarbon ligands, resulting in high aqueous dispersion. The fluorescence intensity of PNCs-PFOS was quenched by TC based on the inner filter effect, in which the excitation spectrum of PNCs-PFOS overlapped with the absorption spectrum of TC. This sensor owned excellent aqueous stability, sensitivity, and selectivity for detecting TC with a LOD of 76 nM [48].

#### 2.2.2. Phospholipid Membrane Coating

**Figure 6:**
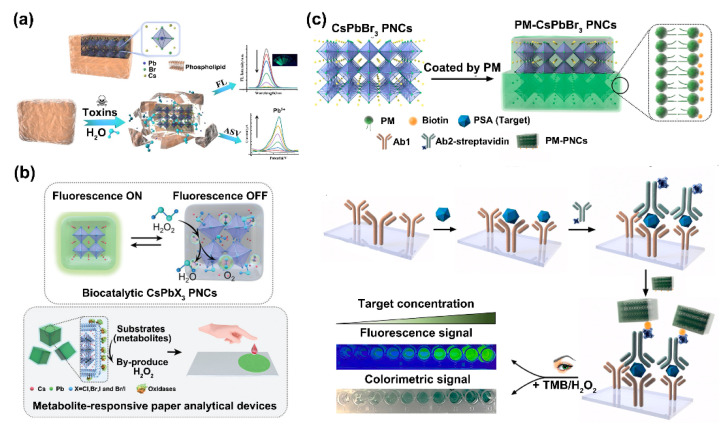
(**a**) Schematic representation of CsPbBr_3_ PNCs@PM for the dual-readout detection of biotoxins. Reprinted from [50], with permission from Elsevier. (**b**) Schematic illustration of the biocatalytic activity of PM-CsPbX_3_ PNCs and the metabolism analysis based on metabolite-responsive paper analytical devices. Reprinted from [51], with permission from John Wiley and Sons. (**c**) Diagram of the synthesis procedures of CsPbBr_3_ PNC nanoprobe and the principle of the dual-readout immunoassay for the detection of PSA. Reprinted from [52], with permission from Elsevier.

Phospholipid is an amphiphilic molecule that consists of a hydrophilic head and a hydrophobic tail. In aqueous solutions, phospholipid molecules can form a membrane by hydrophilic and hydrophobic interaction. Considering the advantages of phospholipid membrane (PM), researchers have used it to improve the water stability/compatibility of PNCs [49]. For instance, Li et al. designed PM-modified CsPbBr_3_ PNCs to construct fluorometric and electrochemical dual-readout assays for broad-spectrum biotoxin (melittin) detection. The outer PM not only served as a shell to maintain the stability of PNCs, but can also react with melittin. Specifically, the melittin-triggered transmembrane pore formation caused water permeation, which broke down the structure of PNCs@PM, generating outstanding fluorescent and electrochemical responses (Figure 6a) [50]. This group also discovered that the encapsulation of CsPbX_3_ PNCs with PM not only greatly enhanced their aqueous stability, but also provided a specific physical environment for enzyme activity study. Consequently, they built a self-reporting probe for metabolism analysis. In this system, CsPbX_3_ PNCs catalyzed the decomposition of H_2_O_2_, the products of which led to rapid fluorescence quenching of the PNCs that were then restored by removing excess H_2_O_2_. As a result, a PM-coated CsPbX_3_ PNC-based paper device was developed which then realized the metabolism analysis via the H_2_O_2_ decomposition induced by the enzyme catalytic reaction (Figure 6b) [51]. Feng et al. developed a PM-coated CsPbBr_3_ PNC-based immunoassay for the fluorescence and colorimetric dual readout detection of prostate-specific antigen (PSA). In addition to greatly improving the aqueous stability of PNCs, the PM coating also helped them resist the unspecific adsorption of biological interferents. Furthermore, biotin-modified lipid was adopted in the outer shell for subsequent surface immobilization. Finally, a sandwich immunoreaction combined with TMB oxidizing reaction was set up for PSA detection (Figure 6c) [52].

### 2.3. Core-Shell Encapsulation for Aqueous Phase Sensing

Compared with the ligand modified on the surface, a dense shell layer may provide PNCs better stability and allow for a wider range of surface functionalization. For example, the core-shell PNC encapsulation methods by using polymer, silica, polystyrene (PS)/silica particles, and metal-organic frameworks (MOFs) were developed to improve the stability and durability of PNCs.

#### 2.3.1. Long-Chain Polymer Encapsulation

**Figure 7:**
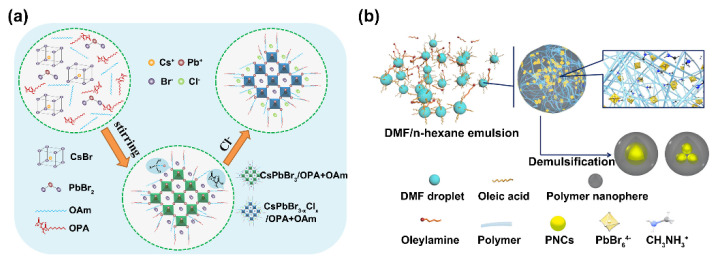
(**a**) Schematic of the synthetic procedure of CsPbBr_3_@OPA+OAm PNCs and the fluorescent colorimetric sensing method for Cl^−^ detection. Reprinted from [55], with permission from American Chemical Society. (**b**) Schematic illustration of the synthesis of PNCs@polymer. Reprinted from [56], with permission from American Chemical Society.

Polymers can be hydrophilic, hydrophobic, or amphiphilic [23,53,54]. Some specific polymers can form protective shells to cover the surface of PNCs and prevent them from degradation. For example, amphiphilic polymers can be used to modify oil-soluble PNCs to make them water dispersive because they contain both hydrophilic groups and multiple hydrophobic units. Compared with traditional small ligands, the long-chain amphiphilic polymer involves multiple hydrophobic units, which have strong interaction with the PNCs to improve their stability. By using long-chain amphiphilic polymers for PNC surface coating, Shu et al. reported a wavelength-shifted colorimetric sensor for the detection of Cl^−^ in sweat based on halogen exchange. In this platform, amphiphilic polymer octylamine-modified polyacrylic acid (OPA) accompanied with oleylamine (OAm) was used as the capping reagent to obtain the highly water-soluble PNCs. It could be found that as the concentration of Cl^−^ increased, the fluorescence emission wavelength of the PNCs shifted from 520 to 441 nm, i.e., the apparent color changed from green to blue. Ultimately, this colorimetry method showed a low LOD of 0.34 mM and obtained high visual resolution (Figure 7a) [55]. In another work by Zhang et al., they developed an inverse emulsion method to synthesize PNCs@polymer nanospheres by using various polymers. In this work, poly(vinylidene fluoride) (PVDF), polystyrene (PS), and poly(methylmethacrylate) (PMMA) were adopted as polymer-protective shells. The PNCs@polymers were endowed with the merits of small size, high color purity, high stability, and good water dispersibility, which were ideal for multidimensional information encryption. In their design, the information to be protected was encrypted in spatial dimension by using uncoated PNCs and PNC@polymers with varied water stabilities. For decryption, after simply spraying water on the paper, the fluorescence of the uncoated PNCs quenched rapidly, while the water-resistant PNCs@polymers remained green fluorescent under UV light. In this way, the encrypted information was translated (Figure 7b) [56].

#### 2.3.2. Silica Encapsulation

**Figure 8:**
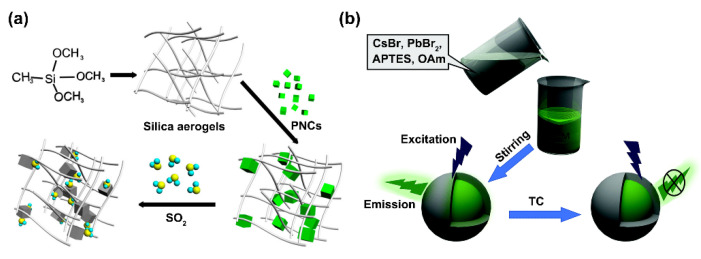
(**a**) Synthesis of PNCs@silica aerogels and the principle of the SO_2_ gas sensor. Reprinted from [59], with permission from American Chemical Society. (**b**) Schematic of the formation of PNCs@silica and the TC sensing. Reprinted from [61], with permission from Royal Society of Chemistry.

Silica shells can be coated on different kinds of nanomaterials to expand their biological applications [57,58]. As for PNCs, Chi et al. developed a novel fluorescence sensing method for SO_2_ gas detection by using silica aerogels-functionalized CH_3_NH_3_PbBr_3_ PNCs as the sensing material. The PNCs@silica had abundant pores, making them suitable for SO_2_ gas sensing and could protect PNCs from being degraded by water. In the absence of SO_2_, PNCs could emit green fluorescence. However, in the presence of SO_2_, a non-emission energy transfer was produced by the coordination reaction between S atoms in SO_2_ molecules and Pb atoms at the surfaces of PNCs, leading to the quenching of PNC fluorescence (Figure 8a) [59]. By using gold nanocrystals (AuNCs)/PNCs@SiO_2_ nanocomposites as the probe, they also reported a visualized ratiometric fluorescence sensor for the detection of Cu^2+^, in which the PNCs@SiO_2_ with green fluorescence was employed as the reference probe, and the AuNCs with red fluorescence was adopted as the sensing probe. With the Cu^2+^ concentration increased in the aqueous solution, the red fluorescence was quenched, whereas the green fluorescence remained stable, causing a fluorescence color variation (orange-red → yellow → green), thus enabling the rapid and visualized detection of Cu^2+^ [60]. Wei et al. proposed a fluorescent sensing platform based on silica layer-modified CsPbBr_3_ PNCs to achieve highly sensitive and highly selective detection of trace TC in ethanol. At room temperature, a silica layer was easily modified by in-situ hydrolysis of 3-aminopropyltriethoxysilane (APTES) on the surface of PNCs without adding water or an initiator in ethanol. It was because APTES caused hydrolysis with traces of moisture present in the air that formed a silica layer to protect the PNCs. When PNCs@silica were in contact with TC, the amino groups on the surface of the silica layer reacted with TC, which gradually quenched the fluorescence of the PNCs. Therefore, the TC content was obtained by detecting the degree of fluorescence quenching (Figure 8b) [61].

#### 2.3.3. PS/Silica Particle Encapsulation

**Figure 9:**
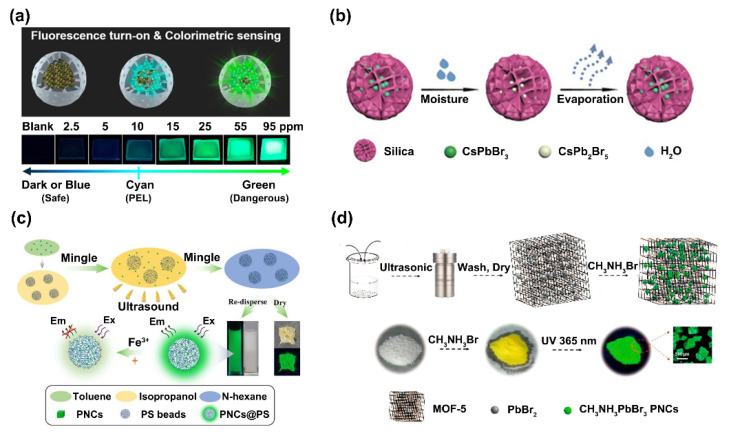
(**a**) The fluorescence turn-on and wavelength-shift dual-mode platform for MA gas sensing. Reprinted from [62], with permission from American Chemical Society. (**b**) Schematic illustration of the reversible transformation process of the CsPbBr_3_ PNCs by removing or exposing them to moisture. Reprinted from [63], with permission from John Wiley and Sons. (**c**) Synthetic scheme diagram of the CsPbBr_3_ PNCs@poly(styrene/acrylamide) (PSAA) composites and the diagram of Fe^3+^ selective sensing. Reprinted from [64], with permission from Elsevier. (**d**) Schematic diagram of the two-step approach for the preparation of CH_3_NH_3_PbBr_3_@MOF-5 composites. Reprinted from [68], with permission from American Chemical Society.

PS/silica particles are widely used in biomedical fields due to their uniform size, easy functionalization surface, monodispersity, and good biocompatibility. Thus, they have the potential to be the hold matrixes of PNCs to allow for their chem/bio sensing applications. Chen et al. reported a fluorescence turn-on and wavelength-shift dual mode sensor for methylamine (MA) gas sensing by using space-confined growth of methylammonium lead tribromide (MAPbBr_3_) PNCs in hollow SiO_2_ nanospheres via the reaction between MA gas and (HPbBr_3_)_2_PbBr_2_@SiO_2_. For the fluorescence turn-on sensing, when the MA gas reacted with (HPbBr_3_)_2_PbBr_2_@SiO_2,_ the PbO byproduct passivated the MAPbBr_3_ PNCs by acting as quantum wells to localize the MAPbBr_3_ PNCs to exhibit quantum-confined optical properties, which would boost the fluorescence intensity. In the wavelength-shift sensing, the red-shift of the fluorescence peak could be attributed to the size increase of the MAPbBr_3_ PNCs formed when the (HPbBr_3_)_2_PbBr_2_@SiO_2_ contacted the MA gas (Figure 9a) [62]. Zhang et al. developed hydrochromic CsPbBr_3_ PNCs for moisture-responsive anti-counterfeiting. When CsPbBr_3_ PNCs were loaded into porous silica, the green emission of the CsPbBr_3_ PNCs@silica reversibly switched on/off by removing or exposing them to moisture, respectively (Figure 9b) [63]. In order to create novel hydrophilic and hyperstatic fluorescent probes for the selective sensing of Fe^3+^ in real samples, Liu et al. adopted a swelling-shrinking strategy to encapsulate CsPbBr_3_ PNCs into poly(styrene/acrylamide) nanospheres. In this design, the fluorescence of the PNCs@PS composites was quenched by Fe^3+^ and the quenching mechanism was inferred to be static quenching (Figure 9c) [64].

#### 2.3.4. MOFs Encapsulation

As an attractive class of porous crystalline materials, metal-organic frameworks (MOFs) have been extensively studied due to their high porosities, tunable pores, and diverse functional sites [65,66]. These unique characteristics make MOFs ideal accommodation for various guest species [67]. Therefore, some researchers have developed PNC-MOF composites to improve the PNCs’ stability. Xia et al. reported a feasible two-step method for synthesizing CH_3_NH_3_PbBr_3_ PNCs embedded in MOF-5. The CH_3_NH_3_PbBr_3_@MOF-5 composites exhibited highly improved thermal stability and water resistance. The composites not only featured excellent temperature-sensing properties with a wide response range from 30 °C to 230 °C, but also exhibited a significant selective fluorescence response to several kinds of metal ions in an aqueous solution. They proposed that the temperature sensing was related to the surface defect states of the PNCs. The possible fluorescence quenching mechanism of Cu^2+^, Al^3+^, Co^2+^, Bi^3+^_,_ and Fe^3+^ was the thermally activated trapping processes involved in the pre-existing trap states. In addition, the fluorescence-enhancing effect of Cd^2+^ could be attributed to the interactions between Cd^2+^ and the organic ligands or coordinated solvent molecules, which strengthened the stability of the composites in an aqueous solution (Figure 9d) [68].

## 3. Perspective

**Figure 10:**
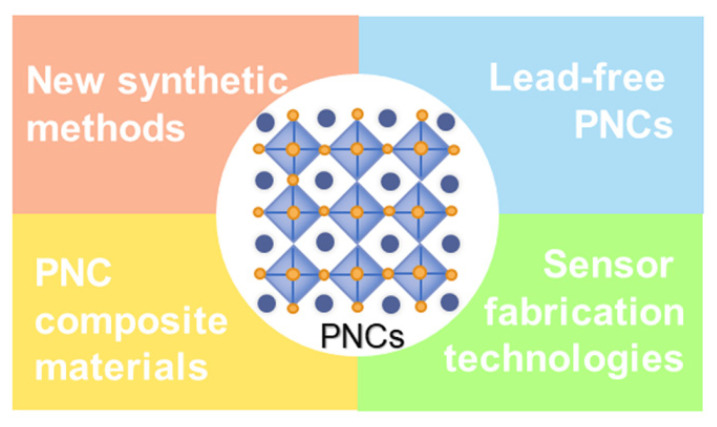
Schematic illustration of the directions that can promote the chem/bio sensing applications of PNCs.

Thanks to the unremitting efforts by researchers, PNCs have become promising materials in the field of chem/bio sensing. In spite of the remarkable progress made in the fabrication of PNCs probes, the chem/bio sensing applications of them are still in infancy compared with those in other fields. Such a situation can be ascribed to the inherent shortcomings of the PNCs, such as the poor stability and the composition of toxic lead ions. What is more, in addition to the protecting strategies introduced in this review, it is also required to develop new technologies to fabricate novel PNCs-based sensors to further improve the stability of the PNCs and simultaneously improve the sensing performance. We therefore believe that further explorations in the flowing directions may promote the prosperity of PNCs in the chem/bio sensing field (Figure 10).

### 3.1. New Synthetic Methods

Regarding the chem/bio sensing applications, the synthetic methods for the construction of PNCs are still in the early stages. Thus, developing new chemistry strategies and more controllable methods to obtain high-performance PNCs is of great significance. For example, Chen et al. synthesized the high-quality CsPbBr_3_ PNCs by a new bromobenzene and alkane amine aliphatic nucleophilic substitution method. They further developed an HCl solution sensor by using this kind of PNCs. With the increasing concentration of HCl, the fluorescence emission wavelength of PNCs blue shifted from 514 nm to 452 nm, resulting in the color changes of the PNCs from green to cyan to blue. Compared with the injection method and ligand-assisted reprecipitation method, this synthetic method had three potential benefits. First, the PNCs were easily obtained in one step without predissolving any precursors, and the growth of PNCs was easily controlled by regulating the aliphatic nucleophilic substitution reaction. Second, the use of low volatility polar solvents that are difficult to remove was avoided, thus the long-term stability of PNCs was improved, which was conducive to sensing applications. Third, the precursors of cesium, lead, and bromide could be separately employed with a tunable ratio. The bromide-rich surface rendered the PNCs immune to successive washing for purification. Furthermore, it also maintained the morphology of PNCs and obtained stable fluorescence at high temperatures. Therefore, this method provided a simpler, more controllable, and reproducible strategy for the synthesis of PNCs [69].

### 3.2. Lead-Free PNCs

Lead-containing PNCs have attracted much attention on account of their exceptional optoelectronic properties. However, the lead content of perovskite materials has raised serious concerns because of its toxicity and accumulation in the ecosystem. Thus, developing an alternative class of lead-free PNCs for chem/bio sensing applications is of great importance.

At present, to avoid the toxicity, researchers have used metals such as copper, indium, and bismuth to substitute for lead. For instance, Revaprasadu et al. found a simple method to synthesize lead-free PNCs (CsCuCl_3_) and used it to selectively detect Pb^2+^ ions. In this sensing, Pb^2+^ could enhance the fluorescence of CsCuCl_3_ due to the chemical interaction between them. Particularly, CsCuCl_3_ revealed promising optical properties with a band gap of 2.6 eV. This kind of PNCs was not only useful as probes for Pb^2+^ but also acted as potential materials for photovoltaic applications [70]. Su et al. reported a novel lead-free perovskite compound (Cs_2_InBr_5_·H_2_O) and used it to detect humidity or traces of water in organic solvents. The novel compound was the first luminescent indium-based perovskite material to be reported, and it featured a unique 0D structure and exhibited red fluorescence with a high PLQY of 33% [71]. Xia et al. discovered a new lead-free metal halide (C_9_NH_20_)_2_MnBr_4_ and developed a highly selective fluorescent sensing platform for acetone vapor detection. This metal halide exhibited a green fluorescence at 528 nm with a high PLQY of 81.08% at room temperature. What is more, (C_9_NH_20_)_2_MnBr_4_ rapidly quenched within 10 s after reacting with acetone vapor, which had no obvious changes with other organic vapors. Thus, (C_9_NH_20_)_2_MnBr_4_ showed outstanding gas sensitivity with high PLQY, quick response, and good selectivity [72]. Song et al. successfully prepared lead-free Cs_3_Bi_2_Br_9_:Eu^3+^ PNCs and employed them for the highly sensitive detection of Cu^2+^ ions in water. The Cs_3_Bi_2_Br_9_:Eu^3+^ PNCs demonstrated multicolor emissions including the emission of the PNCs and the ^5^D_0_−^7^F_J_ transition for Eu^3+^ ion. Compared to the bare Cs_3_Bi_2_Br_9_ PNCs, the Eu^3+^-doped PNCs achieved excellent water stability, higher PLQYs (≈42.4%), and multicolor emissions including red light. Furthermore, the PNCs showed an outstanding probing ability for Cu^2+^ ions with high selectivity in water, which demonstrated a wide detection range from 5 nM to 3 μM and a LOD of 10 nM [73].

### 3.3. Develop PNCs Composite Materials

Developing hybrid nanomaterials is an efficient method to broaden the optical properties and stability of PNCs. For example, Zhang et al. developed a one-pot synthesis method to obtain watermelon-like PNC-upconversion nanoparticle (UCNP) hybrid composites consisting of cubic-phase PNCs and hexagonal-phase UCNPs by using cubic phase UCNPs as the intermediate transition material. The composites were NIR-excitable with much-improved stability compared to the conventional PNCs. It is believed that with the development of synthesis technology, the hybridization of PNCs with other nanomaterials will pave new ways for the chem/bio sensing application of PNCs [74].

### 3.4. New Sensor Fabrication Technologies

Aside from the inherent features of the PNCs such as their morphology, chemical composition, and optical properties, it is generally acknowledged that the post-synthesis fabrication is a critical way to maintaining the stability of the PNCs and endowing them with target specificity for chem/bio sensing applications. Therefore, in addition to the surface protection strategies aforementioned, emerging methods to fabricate facile PNC-based sensors are highly desired in this area. For example, molecularly imprinted polymer (MIP) and electrospun fiber membrane coatings are two representative PNC sensor fabrication strategies.

MIPs, also named artificial antibodies, are synthetic materials that own specific cavities that are complementary to templates. These artificial tailor-designed materials are able to specifically recognize the targets. Therefore, MIPs have been widely used in sensing applications. On account of the eminent recognition ability of MIPs, as well as the protection they can provide the PNCs, the integration of MIPs with PNCs is a promising strategy to obtain superior PNCs-based sensors. As an example, Liang et al. designed a novel CsPbBr_3_ PNCs@MIPs sensor for highly specific and sensitive recognition of omethoate (OMT). In this sensing, the OA-capped PNCs had carboxylic groups on the surface that could react with tetramethylorthosilicate (TMOS) and APTES to eventually form MIPs-coated PNCs. It is worth noting that APTES could absorb trace moisture in the air and hydrolyze to form a silica matrix-protective shell to protect the PNCs. Due to the presence of the specific cavities in the PNCs@MIPs composites, they had a highly specific binding toward OMT, leading to the fluorescence quenching based on the charge transfer from the PNCs to OMT [75]. In another example, Wei et al. reported a sensitive and selective fluorescent sensor by utilizing CsPbBr_3_ PNCs@MIPs as probes for trace TC detection in aqueous environments. When TC was bound to the imprinting cavity on the surface of the PNCs@MIPs, the electron transfer between them caused fluorescence quenching of the PNCs@MIPs [76]. Liang et al. constructed a highly sensitive and selective method for phoxim assay by using novel CsPbBr_3_@MIPs composites. The PNCs were encapsulated in a silica matrix MIP layer via a sol-gel method that slowly hydrolyzed the organosilicon monomers in situ. The specificity of the assay was originated from the imprinted cavities complementary to phoxim [77].

The electrospinning technique has been found have a high performance and cost-effective technology for fabricating large surface area electrospun fibrous membranes for numerous sensing applications. The large surface area of the fiber membrane shows the potential to provide remarkably high sensitivity and fast response time in chem/bio sensing applications. In recent years, researchers have combined electrospinning technology with PNCs to improve the stability of PNCs and fabricate sensors. Li et al. prepared PS fiber membrane-encapsulated CsPbBr_3_ PNCs through the electrospinning method, and displayed its extraordinary stability in aqueous and ethanol media for the ultrasensitive detection of rhodamine 6G (R6G) by means of fluorescence resonance energy transfer (FRET). The composite combined the optical properties of PNCs and the stabilizing capacity of the PS fiber membrane, showing a remarkable LOD of 0.01 ppm [78]. Afterward, this group used an electrospinning method to encapsulate CsPbBr_3_ PNCs into a PMMA fiber membrane and applied it to the fluorescence detection of trypsin, Cu^2+^, and pH after further surface functionalization [79].

It should be noted that the above-mentioned trends cannot represent all the promising directions to promote the chem/bio sensing applications of PNCs. We strongly believe that there are more opportunities in this field.

## 4. Summary

The high PLQY, color purity, and wide absorption spectra of PNCs make them very attractive for chem/bio sensing. In this review, the current chem/bio sensing applications of PNCs were summarized. We also discussed the new technologies covering from new nanofabrication to developing trustable sensors that may lead the future trend for the chem/bio sensing applications of PNCs. We believe that the consistent effort devoted to this area will put the PNC-based sensing methods into practical applications in the near future.

## Data Availability

Not applicable.
